# Determination of hexachlorophene residue in fruits and vegetables by ultra-high performance liquid chromatography-tandem mass spectrometry

**DOI:** 10.1371/journal.pone.0307669

**Published:** 2024-08-14

**Authors:** Yuan Ma, Yan Chen, Chaozheng Wang, Dandan Li, Kaizhi Xuan, Zhengfeng Lin, Jiahan Wang, Zihao Su, Yuexian Wu

**Affiliations:** 1 The Food Testing Center, Key Laboratory of Tropical Fruits and Vegetables Quality and Safety for State Market Regulation, Hainan Inspection and Testing Research Institute, Haikou, China; 2 Technology Center of Haikou Customs District, Haikou, China; 3 Haikou Marine Geological Survey Center of China Geological Survey, Haikou, China; 4 Hainan Medical University, Haikou, China; Center for Research and Technology Transfer, VIET NAM

## Abstract

A modified QuEChERS (Quick, Easy, Cheap, Effective, Rugged and Safe) -LC-MS/MS method was developed for the determination of hexachlorophene in fruits and vegetables. Samples were extracted by acetonitrile and then salted with an acetate buffer system. Extractants neutral alumina (Al-N), strong cation exchange silica gel bonded adsorbent (SCX) and graphitized carbon black (GCB) were used for sample purification. The method demonstrates excellent accuracy and reproducibility. Under optimized conditions, the correlation coefficients of hexachlorophene were higher than 0.995 in the range of 0.5–20 ng/mL. The limit of quantification (LOQ) was 2.0 μg/kg. The average recoveries, assessed at three spiked levels (2.0, 4.0, and 20.0μg/kg) across various matrices including cabbage, celery, tomato, eggplant, potato, radish, cowpea, chives, apple, peach, grape, citrus, bitter melon, banana and hami melon ranged from 72.0 to 100.5% with relative standard deviations from 3.2 to 9.8% (n = 6).

## Introduction

Hexachlorophene, a broad-spectrum bactericide, has a wide range of applications, including in consumer antibacterial products, agricultural products, veterinary medicine and cleaning products [1–4]. Hexachlorophene is a highly lipophilic chlorinated bisphenol, which have the potential to contaminate aquatic ecosystems, thereby posing risks to human health through the food chain [5], according to GB2763-2021 in China, the acceptable daily intake (ADI) of hexachlorophene was 0.0003 mg/kg bw. Studies have shown that several cases of human poisoning of hexachlorophene, daily doses of 20 mg/kg taken for three days cause toxic symptoms in children, [6] and more than 30 children died due to the use of a talcum powder containing an excess 6% hexachlorophene in France [7]. Chinese and European Union (EU) cosmetic hygiene regulations prohibit the use of hexachlorophene as a preservative and disinfectant in cosmetics. The latest version of China’s maximum residue limits for pesticides (GB2763-2021-China) has added allowable levels for hexachlorophene residues in foods (the maximum residue limit, MRLs).

In terms of literature reports, gas chromatography (GC) method [8], gas chromatography-mass spectrometry (GC-MS) method [9], liquid chromatography (LC) method [10],capillary electrophoresis (CE)method [11] and liquid chromatography-tandem mass spectrometry (LC–MS/MS) method [12, 13]were mainly used to analyze the content of hexachlorophene in skin, sewage sludge, cosmetics, tea or agricultural products. When hexachlorophene is analyzed directly by GC or GC-MS, the detection sensitivity is low, and derivatization of hexachlorophene is necessary to increase the sensitivity, which is complex and time consuming. Among them, LC–MS/MS method is the most selective and sensitive technique for identification and quantification of hexachlorophene.

The currently reported methods for the detection of hexachlorophene residues in food mainly use the QuEChERS method combined with liquid chromatography-tandem mass spectrometry (LC-MS/MS) [12, 13]. The simple and efficient pre-treatment combined with the specificity and high sensitivity of tandem mass spectrometry, which can meet the requirements of maximum residue limits. For multi-residue pesticides detection, primary-secondary amine adsorbent (PSA) and graphitized carbon black (GCB) are mostly used in the literatures [14–16] and standard methods (AOAC 2007.01, EN 15662:2008 and GB 23200.121–2021), unfortunately, they can adsorb phenolic compounds with benzene ring and hydroxyl functional groups during the purification process [17, 18], which lead to a target loss. When analyzing diverse food types, the optimal quantities of primary secondary amine (PSA) and graphitized carbon black (GCB) must be adjusted according to the specific matrix. This requires substantial effort to determine the ideal adsorbent levels for various food matrices, a challenge that hinders the establishment of standardized hexachlorophene detection methods. For instance, the purification processes for apple and tea samples show considerable differences, emphasizing the need for tailored approaches in purification techniques [13].

Fruits and vegetables are important components of the current diet of the general population, but a standard method for the determination of hexachlorophene residues in fruits and vegetables has not yet been established. In this study, a modified QuEChERS -LC-MS/MS method was developed for the determination of hexachlorophene pesticide residues in fruits and vegetables. Conditions of pre-treatment, chromatography, and mass spectrometry were optimized. Fifteen kinds of fruits and vegetables, including chives, cabbage, celery, tomato, potato, apple, grape and others were selected as representative matrixes to verify the effectiveness of the method. The aim of this article is to establish a highly efficient, rapid, accurate and reliable method for the determination of hexachlorophene pesticide residues in fruits and vegetables, which can provide a reference for the establishment of detection standards which could meet the requirements of pesticide residue limit regulations.

## Materials and methods

### Instruments

TQ-S ultra-high performance liquid chromatography-mass spectrometry (UPLC-MS/MS) (USA Waters Corporation); DMV-16 vortex mixer (Guangdong Keyin Company); Centrifuge 5810 R centrifuge (Eppendorf Company); MS204TS electronic balance (Swiss Mettler Toledo Company).

### Reagents and materials

Hexachlorophene (98.7% purity) was sourced from Dr. Ehrenstorfer (UK). High-performance liquid chromatography (HPLC)-grade solvents, including methanol, acetonitrile, ammonium acetate, and formic acid, were procured from TEDIA (USA). Anhydrous magnesium sulphate was acquired from Sinopharm Chemical Reagent Co., Ltd (China). QuEChERS extraction salt packets, each containing 4 grams of anhydrous magnesium sulfate, 1 gram of sodium chloride, 1 gram of disodium citrate dihydrate, and 0.5 grams of disodium citrate hydrate, were obtained from Shanghai Supu (China). The sorbent materials including positive phase silica gel adsorbent (Sillca), strong anion exchange silica gel bonded adsorbent (SAX), strong caution exchange silica gel bonded adsorbent (SCX), neutral alumina (Al-N), acidic alumina (Al-A), basic alumina (Al-B), positive phase silica bound amino adsorbent (NH2), graphitized carbon black (GCB), primary-secondary amine (PSA), reverse phase silica bonded ethyl adsorbent (C2), reverse phase silica bonded octyl adsorbent (C8), reverse phase silica bonded octadecyl adsorbent (C18), reverse phase silica bonded cyanoethyl adsorbent (CN), polystyrene/divinylbenzene adsorbent (PLS), reverse phase silica bonded phenyl adsorbent (PH) were all purchased from Dima Technology Co., Ltd. (China). Deionized water with 18.2 MΩ cm resistivity was produced using the laboratory’s ultrapure water system from Millipore (USA). Nylon66 syringe filters each with a 0.22 μm pore size, were acquired from Tianjin Jinteng (China).

### Samples

The 15 types of fruits and vegetables including cabbage, celery, tomato, eggplant, potato, radish, cowpea, chives, apple, peach, grape, citrus, bitter melon, banana and hami melon were all purchased from local large supermarkets in Haikou City. These samples were tested and confirmed to have no detectable levels of hexachlorophene. The edible portions were homogenized, then placed into polyethylene bottle, and stored at -18 °C. Before pretreatment, the frozen samples were allowed to thaw completely at room temperature.

### Standard solutions

To prepare a 1,000 mg/L stock solution, 10.13 mg hexachlorophene was dissolved in 10 mL of acetonitrile, and stored at -18 °C in a dark environment. Working solutions were obtained by diluting the stock standard with acetonitrile to the required concentrations. A 1,000 μg/L working solution was utilized for the optimization and validation of the method. Blank samples were processed as described in the “Sample Pretreatment” section to yield a blank matrix solution. The working solution was gradually diluted with blank matrix solutions to prepare a series of matrix-matched standard curve solutions.

### Optimization of LC-MS/MS analysis

A 1,000 μg/L hexachlorophene standard solution was automatically sent into the mass spectrometer. The molecular formula of hexachlorophene was inputted, and the IntelliStart function of MassLynx was employed to automatically optimize the ionization parameters and ion pairs. The optimized conditions were shown below:

1. Electrospray negative ion source (ESI^-^);
2. Capillary voltage: 3.0 kV;
3. Ion source temperature: 150 °C;
4. Desolvation temperature: 350 °C;
5. Nebulizer gas: 150 L/hr nitrogen;
6. Drying gas: 650 L/hr nitrogen;
7. Collision gas: argon;
8. Scanning mode: multiple reaction monitoring (MRM) mode;
9. Quantitative and qualitative ion parameters were given in Table 1.

**Table 1:** Ion mass spectrometry parameters of hexachlorophene.

Chemical compound	Quantification/Qualification Ion(m/z)	Cone (eV)	Collision(eV)	Dwell Time(ms)
Hexachlorophene	402.9>194.9 (Quantification)	42	28	50
	402.9>366.9 (Qualification)	42	22	50

The analytes were separated by a non-polar BEH C18 column (2.1×50mm, 1.7 μm) from Waters, flow rate: 0.35 mL/min; injection volume: 3μL; column temperature: 30 °C; mobile phase: Phase A is methanol and Phase B is 0.05% formic acid water solution (containing 10 mmol/L ammonium acetate). The gradient elution program was as follows: 0~2.0 min, 10% A; 2.0~2.2 min, 10% A to 95% A; 2.2~5.0 min, 95% A; 5.0 min~5.1 min, 95% A to 10% A; 5.1 min~5.5 min, 10% A.

### Adsorption experiments for standard solution by different adsorbents

10 mg of the GCB adsorbent and 100 mg of other adsorbents were weighed in same tubes, respectively. then a 4 mL of 10 ng/mL standard solution were added to the tubes, after uniformly mixed and centrifuged, 1 mL of supernatant was moved to a clean vial for determination. 3 duplicates were applied and compared with a 10 ng/mL standard solution without any preparation to calculate the recovery.

### Sample preparation

5g of the homogenized samples were weighed and placed into a 50 mL centrifuge tube. 5 mL of water were added and whirled by a vortex mixer for 1 min, 10 mL of acetonitrile were added and whirled for 3 min and the QuEChERS extraction salt package were added and whirled for 2 minutes, then centrifuged at 9000 rpm for 5 minutes. Then 4 mL of the supernatant were transferred into a clean-up tube (15 mL) containing 600 mg anhydrous magnesium sulfate and the following sorbents. (1) 100 mg PSA; (2) 10 mg GCB; (3) 100 mg PSA+10 mg GCB; (4) 100 mg Al-N; (5) 100 mg SCX; (6) 100 mg Al-N 100 mg SCX, respectively. The mixture was shaken for 1 min and still standing for 5 min, at last 1 mL of the supernatant was filtered into a clean vial by a 0.22 μm Nylon66 syringe filter for analysis.

### Method validation

Method validation was performed according to the guidelines reported in SANTE/11312/2021 (European Commission, 2021). The analytical parameters evaluated were linearity, limits of quantification (LOQ) and limits of detection(LOD), matrix effect, mean percent recovery (RE%) as a measure of trueness, intra-day precision as repeatability (RSD) and selectivity.

The standard curve was prepared using acetonitrile or blank matrix extracts solution at the levels of 0.2, 0.5, 1.0, 2.0, 5.0, 10.0, 20.0 ng/mL. The peak area of the quantitative ion was plotted as the vertical axis and the mass concentration X (ng/mL) as the horizontal axis to create the standard curve. Method linearity was defined by the coefficient of determination (r) of the calibration curves.

The detection and quantification limits of this method were determined by using matrix-spiked samples. The detection limit was calculated by a signal-to-noise ratio of 3, and the quantification limit was calculated by a signal-to-noise ratio of 10 according to the qualitative and quantitative ions, based on actual sample experiments, the highest value obtained from the 15 matrices was defined as the method’s detection limit.

The matrix effect, expressed as a percentage matrix factor (ME), was calculated by comparing the differences in the response of the standard in matrix extract and in solvent according to the following equation:

$$ME\left(\text{\%}\right)=\left(\frac{Rstd\;matrix\;extract}{Rstd\;solvent}-1\right)\times 100$$

Rstd matrix extract = peak area of the standard in the matrix extract.

Rstd solvent = peak area of the standard in the solvent.

Recovery experiments were conducted in 15 blank matrices, including cabbage, celery, and tomato, among others, to investigate the accuracy and precision of the method. The spiked levels were 2.0, 4.0, and 20.0 μg/kg, and each spiked level was applied in 6 duplicates. Matrix-matched calibration and external standard method were used for quantification.

## Results and discussion

### Selection of a gradient elution program

This experiment aims to investigate the effect of ammonium acetate and formic acid to the mobile phase on the mass spectrometry signal. Two mobile phase systems were compared: water-acetonitrile and water-methanol. It was found that when water-methanol was used as the mobile phase, the peak shape and response of hexachlorophene was significantly better than when water-acetonitrile was used. On this basis, the effects of methanol-water, methanol-0.05% formic acid in water and methanol-0.1% formic acid in water on the chromatographic peaks were investigated. It was observed that the addition of formic acid increased the mass spectrometric response, with the highest response being obtained using 0.05% formic acid solution. The effect of 0.05% formic acid in water and 0.05% formic acid with 10 mmol/L ammonium acetate as mobile phase on the chromatographic peaks was also compared. The results showed that the addition of ammonium acetate to the mobile phase increased the response of hexachlorophene. Therefore, the final mobile phase system used was an organic phase of methanol and an aqueous phase containing 10 mmol/L ammonium acetate and 0.05% formic acid in water. The chromatograms of hexachlorophene using different mobile phases are shown in S1 Fig.

### Investigation of clean-up methods

**Fig 1:**
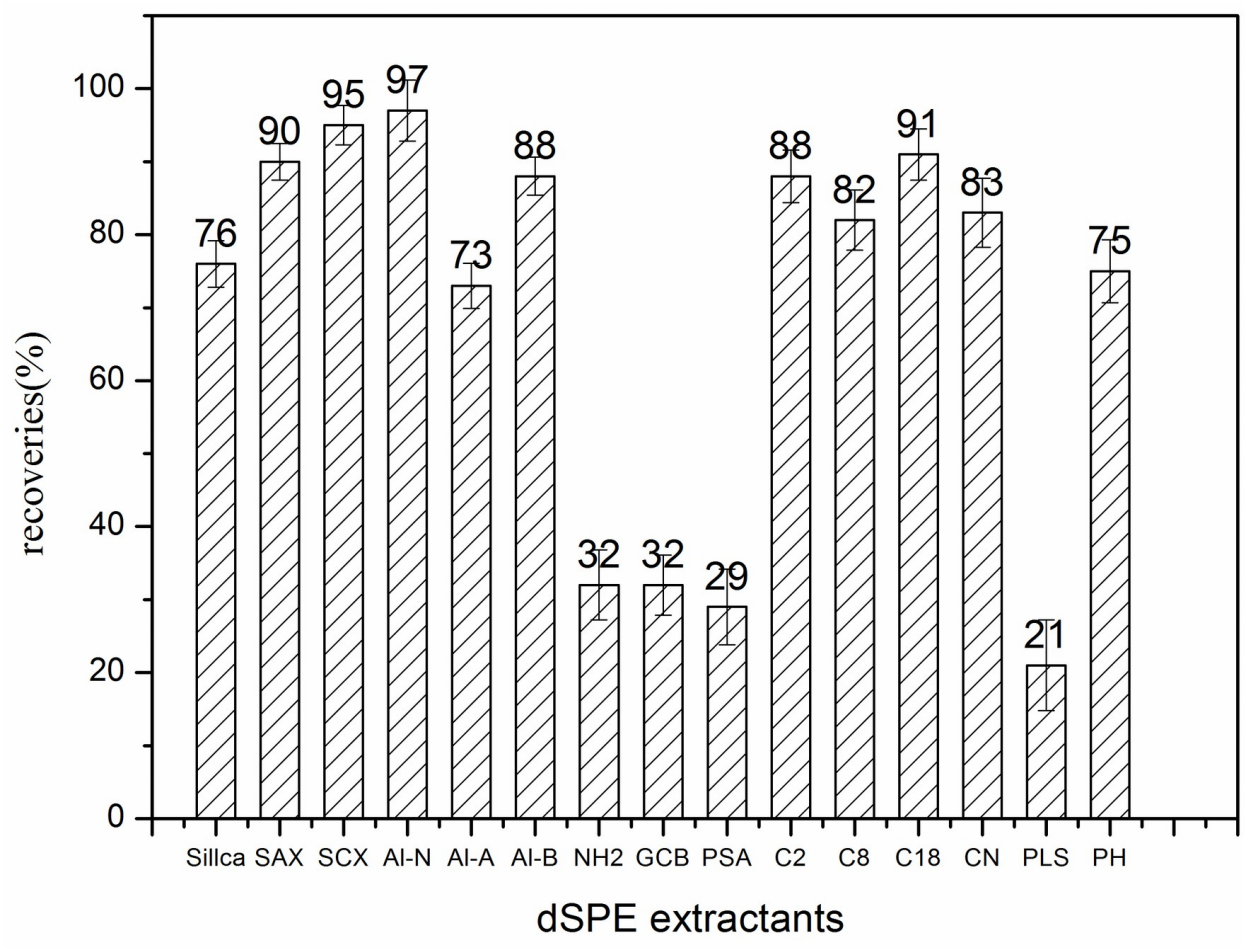
Recoveries of standard solution after adsorption experiment with different adsorbent.

Fig 1 and S1 Table illustrates the recoveries of 10 ng/mL standard solution following the adsorption experiment. The recoveries were found to be lower than 50% after PSA, GCB which are currently used in standard methods (AOAC 2007.01, EN 15662:2008 and GB 23200.121–2021). This indicates that these purification materials have strong adsorption capabilities for the target compounds. GCB can remove pigment components from the extract, but it strongly adsorbs compounds that contain benzene ring functional groups [17, 18]. On the other hand, PSA primarily adsorbs phenolic acid impurities in the matrix. This may explain the lower recoveries of these two materials for hexachlorophene. Zheng et al. [13] also found that PSA and GCB can adsorb hexachlorophene, and the adsorbent effect depends on different matrices. The Al-N and SCX have the highest recoveries, which exceeded 90%. Neutral alumina is capable of adsorbing impurities, including pigments [19], while SCX exhibits strong cation exchange performance. Additionally, due to the presence of benzene rings, it can also adsorb non-polar substances [20].

Then we investigated the recoveries of different materials after adsorption using potatoes, tomatoes, and celery as typical matrices. In this study, the recoveries of six different combinations of adsorbents were compared. To ensure accuracy, working standard solutions were added to the blank samples to reach a concentration of 10 μg/kg for each analyte. The results in Table 2 and S2 Table indicate that the recoveries of PSA and GCB were below 80% when potatoes and tomatoes were used as matrices. However, in the case of celery, the recovery was higher than 70% and significantly greater than that in potatoes and tomatoes. This difference may be attributed to the competitive adsorption effect [21]. When the sample contains more impurities, the purification material selectively adsorbs them, resulting in a decrease in the adsorption of hexachlorophene. Zheng et al [13] also found that significant differences in the adsorption losses of phenolic pesticides in tea and Chinese cabbage matrices with different PSA and GCB contents. This suggested that the optimal amount and ratio of PSA and GCB for purification need to be optimized for different matrices, which hindered the standardization of hexachlorophene detection in various fruits and vegetables. When purifying using Al-N and SCX, all three matrices had a recovery greater than 80%, and there was no significant difference in the impact of the matrix on the recovery rate. However, when using Al-N and SCX for dispersive solid-phase extraction, the removal of pigments from dark colour samples such as celery still needs improvement.

**Table 2:** Recoveries of three matrix clean-up with different d-SPE adsorbents.

Sample matrix	Recovery(%)±SD					
	PSA	GCB	PSA+GCB	Al-N	SCX	Al-N+ SCX
Potato	43.2±7.2	68.3±4.2	26.4±7.0	93.2±2.9	95.4±4.2	95.1±2.3
Tomato	78.2±5.0	72.4±3.6	47.3±4.6	91.3±2.8	97.2±3.1	96.3±2.8
Celery	89.2±4.1	92.1±2.2	72.3±5.3	89.6±3.8	96.3±4.5	87.1±3.7

**Fig 2:**
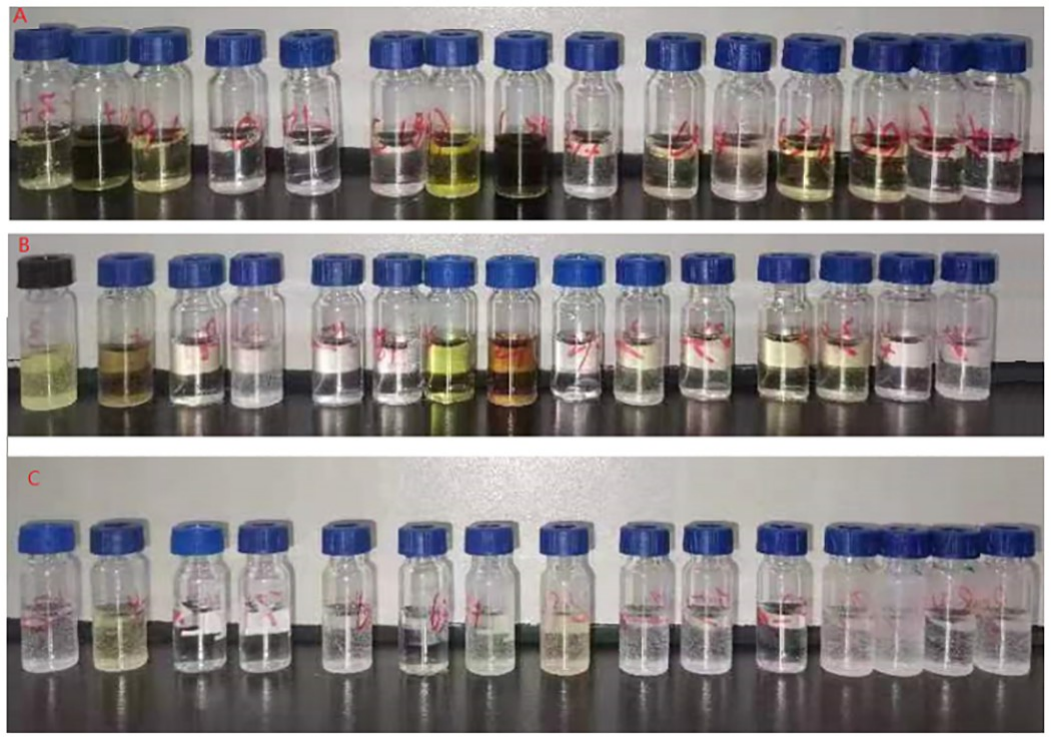
Sample colors of extract samples: (A) Unpurified; (B) Purified with Al-N+SCX; (C) Purified with Al-N+SCX+GCB (From left to right, the order is 1. Cabbage, 2. Celery, 3. Tomato, 4. Eggplant, 5. Potato, 6. Radish, 7. Cowpea, 8. Chives, 9. Apple, 10. Peach, 11. Grape, 12. Citrus, 13. Bitter Melon, 14. Banana, 15. Hami Melon).

A comparison was conducted between the purification effects and recoveries of 15 kinds of fruits and vegetables using two purification methods: Al-N+SCX and Al-N+SCX+GCB. The working standard solutions were added to the blank samples to achieve a concentration of 10 μg/kg for each analyte. The results presented in Fig 2 and Table 3 demonstrated that the addition of GCB for purification was enabled in dark- colored samples, such as celery and chives, to obtain clear extracts with recoveries greater than 70%. However, for samples such as eggplant and radish, adding GCB for purification resulted in significant losses in recoveries. Dark-colored samples contain more interfering substances, which results in a weaker adsorption capacity of GCB for hexachlorophene, leads to better recovery rates.

**Table 3:** Comparison of recoveries of various matrices after two purification treatments.

No.	Matrix	Recovery(%)		Matrix effect(%)			The selected plan
		A*	B*	No purification	A*	B*	
1	Cabbage	88.2	45.3	-20.4	-19.6	-9.0	A*
2	Celery	89.3	87.2	-35.2	-16.1	-10.4	B*
3	Tomato	81.7	60.2	-26.1	-16.5	-6.2	A*
4	Eggplant	77.6	52.3	-18.9	1.2	-4.6	A*
5	Potato	90.8	56.4	-12.6	1.2	2.4	A*
6	Radish	91.2	60.2	-24.3	-0.5	-3.8	A*
7	Cowpea	87.7	82.6	-32.6	-15.7	-10.3	B*
8	Chives	86.4	83.5	-57.9	-43.3	-25.8	B*
9	Apple	98.3	70.2	-14.3	-6.2	-2.2	A*
10	Peach	86.2	65.3	-3.7	-5.8	-1.1	A*
11	Grape	91.6	72.1	13.4	-1.3	4.5	A*
12	Citrus	88.4	87.7	-21.2	-7.0	2.8	B*
13	Bitter Melon	92.3	82.4	-24.6	-15.7	-9.4	B*
14	Banana	85.5	62.5	-8.4	-11.2	-1.5	A*
15	Hami Melon	82.3	35.4	8.0	2.2	4.5	A*

*A purification by Al-N+ SCX

*B purification by Al-N+ SCX+GCB

It is known that the presence of undesired impurity substances may affect the ionization process which brought strong matrix effects [22]. Table 3 demonstrates that the matrix effects for the majority of samples were notably diminished following purification with Al-N+SCX, as compared to the unpurified sample solutions. This suggests that a significant quantity of interfering substances can be effectively removed. However, chives had the strongest matrix effect (-43.4%) which are a complex matrix in pesticide residue analysis [23, 24]. After purification with Al-N+SCX+GCB, the matrix effect of chives significantly reduced and other matrices was further reduced. The ME value of most matrices were lower than ±20% and considered not to have significant ME except chives (-25.8%). Thus, the final experimental protocol was established as follows: Al-N and SCX were used for dispersive solid-phase extraction to eliminate impurities without adsorbing hexachlorophene for most samples. For dark-colored samples, Al-N+SCX+GCB were used for dispersive solid-phase extraction. The final purification selection for the 15 matrices is presented in Table 3.

### Method validation

The linear equations and correlation coefficient r values in the range from 0.2–20 ng/mL were shown in S3 Table, acceptable linearity was obtained for both considering the solvent and matrix-match calibration curves with a correlation coefficient>0.995.

**Fig 3:**
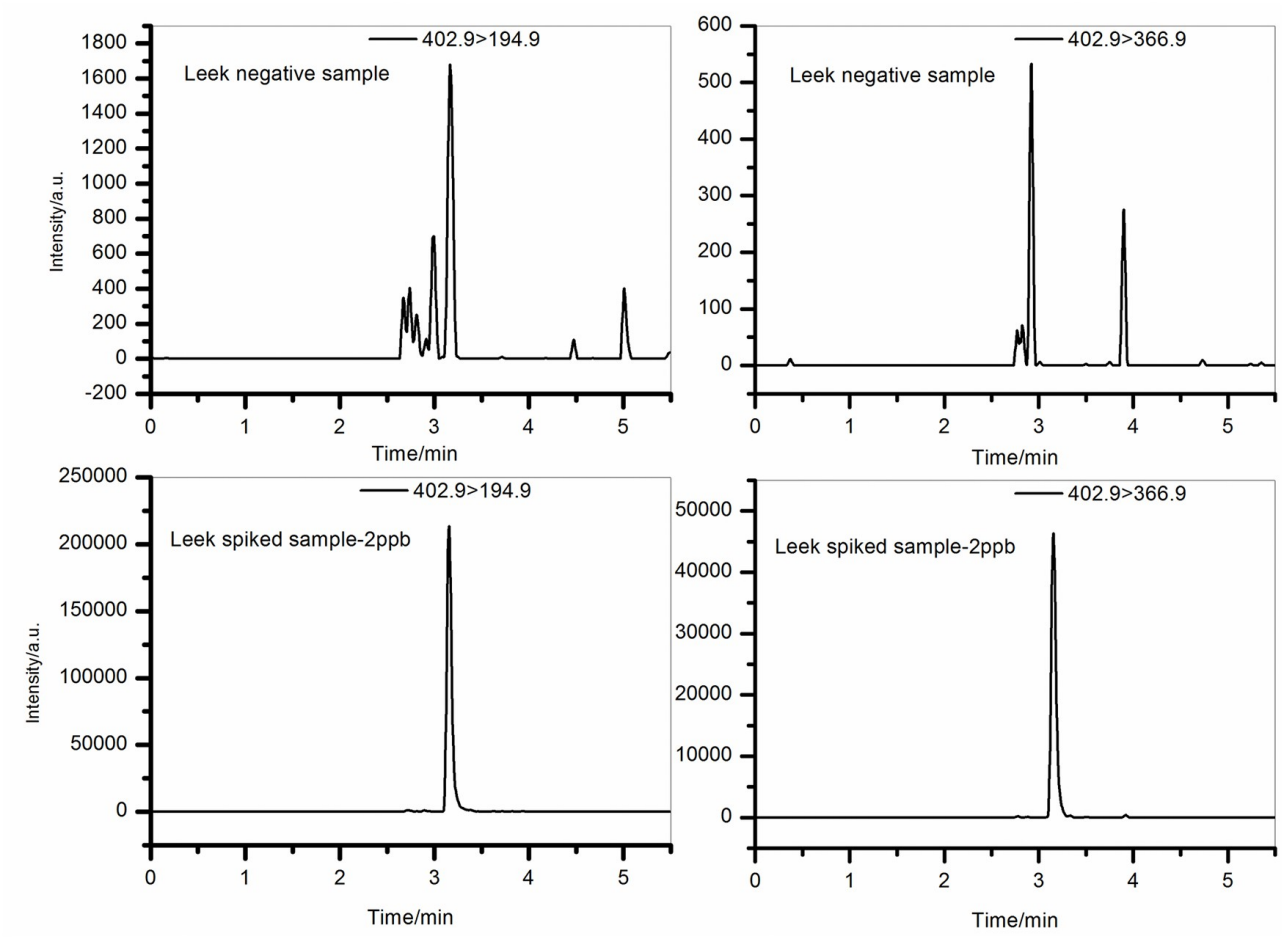
MRM image of blank chive sample and its spiked sample (spiked with 2.0 μg/kg).

The detection and quantification limits of this method were determined by using matrix-spiked samples. Based on actual sample experiments, the highest value obtained from 15 matrices was defined as the method’s detection limit. The detection and quantification limits for hexachlorophene were 0.6 μg/kg and 2.0 μg/kg, respectively, meeting the maximum residue limit requirements (0.01 mg/kg) specified in GB 2763–2021. Fig 3 shows the spectra of blank chives samples and corresponding spiked samples at the quantification limit concentration.

Recoveries were determined by spiking 15 kinds of fruits and vegetables samples with 2.0, 4.0, and 20.0 μg/kg following the optimized sample preparation conditions. Table 4 summarizes the recoveries and precision, and the results of each spiked recovery were shown in S4 Table. Table 4 shows that the relative standard deviation (RSD) of hexachlorophene was between 3.2% and 9.8%, and the average recovery was between 72.0% and 100.5% under three spiked levels in various fruit and vegetable matrices. The results indicated good recoveries for the proposed assay (70–110%) with the relative standard deviation less than 10%.

**Table 4:** The recoveries and relative standard deviations of hexachlorophene in 15 kinds of fruits and vegetables (n = 6).

Matrix type	Spiked level					
	2.0 μg/kg		4.0 μg/kg		20.0 μg/kg	
	Recovery (%)	RSD (%)	Recovery (%)	RSD (%)	Recovery (%)	RSD (%)
Cabbage	88.5	9.2	85.7	6.3	89.4	4.2
Celery	85.4	9.5	84.7	9.8	83.5	3.9
Tomato	81.2	7.3	80.2	5.5	78.7	3.2
Eggplant	75.8	8.4	73.5	5.9	77.4	3.8
Potato	89.2	7.7	87.8	5.9	88.9	4.3
Radish	92.3	9.3	92.8	8.2	92.5	7.6
Cowpea	84.6	8.8	88.1	8.1	85.0	4.6
Chives	82.8	7.5	81.8	7.2	82.1	5.8
Apple	98.2	9.3	96.8	4.2	100.5	5.2
Peach	86.7	8.6	85.3	6.4	86.1	4.4
Grape	88.4	8.9	91.6	7.8	85.6	4.2
Citrus	73.5	9.6	72.0	7.5	77.4	7.8
Bitter melon	80.8	8.6	81.4	7.3	83.5	7.5
Banana	83.5	8.2	90.9	6.2	81.1	6.5
Hami Melon	78.6	9.5	81.7	6.9	80.7	5.5

### Comparison to other methods

The comparison of some references about determination of hexachlorophene was shown in Table 5. Compared with previously reported methods, the proposed method determined by LC-MS/MS has an advantage in LOQ over other equipment. The developed method demonstrated that recoveries were acceptable for15 kinds of fruits and vegetables matrixes, additionally, our results showed that natural matrix of fruits and vegetable can influence the recoveries when d-SPE cleanup by PSA.

**Table 5:** Comparison of proposed method respect to references published by others.

No.	Matrix	Equipment	Sample preparation	LOQ	Recovery(%)	Reference
1	black tea,	LC-MS/MS	QuEChERS-dSPE cleanup with C18、GCB 、PSA	0.17μg/kg	76.9~113.2	[13]
	soybean,					
	apple, and					
	pakchoi					
2	human adipose tissue	GC-ECD	Acid-base partition cleanup and alkali derivatization	10μg/kg	96	[26]
3	Cosmetics	HPLC	ionic liquid dispersive liquid—liquid microextraction followed by magnetic solid-phase extraction	0.46μg/mL	74.5~97.7	[10]
4	tomatoes, sweet corn, cucumbers, and milk	GC-ECD	cleanup on a Celite-H2S04 column and alkali derivatization	20μg/kg	67~137	[25]
5	Cosmetics	capillary electrophoresis	ultrasonically extracted	0.19μg/mL	90~96.4	[11]
6	15 kinds of fruits and vegetables	LC-MS/MS	QuEChERS-dSPE cleanup with Al-N、SCX、GCB	2.0μg/kg	72.0~100.5	This work

## Conclusions

Recently, limits for hexachlorophene residues were established in GB2763-2021-China; however, corresponding detection methods have not yet been established. Therefore, it is necessary to establish a reliable method to regulate food safety. Adsorbents PSA using in the conventional QuEChERS method have strong adsorption capabilities for hexachlorophene, and difficult to apply to various fruits and vegetables. This study describes a method for detecting hexachlorophene residues in fruits and vegetables using UPLC-MS/MS. The pretreatment involves using SCX, Al-N, and GCB sorbents for dispersion-extraction. The method meets the requirements for methodological evaluation in terms of accuracy and precision, and the quantitative limit satisfies the GB 2763 limit for hexachlorophene in fruits and vegetables. The method is rapid, efficient, sensitive, stable, and reliable. It is expected to provide a basis for establishing relevant detection standards.

## Supporting information

S1 Fig TIC image of hexachlorophene standard solution using different mobile phases. (A) acetonitrile-water, (B) methanol-water, (C) methanol-0.05% formic acid in water, (D) methanol-10 mmol/L ammonium acetate and 0.05% formic acid in water.
(PDF)

S1 Table Recoveries of standard solution after adsorption experiment with different adsorbent. (PDF)

S2 Table Recoveries of three matrix clean-up with different d-SPE adsorbents. (PDF)

S3 Table The linear equations and correlation coefficient values of hexachlorophene. (PDF)

S4 Table The recoveries and relative standard deviations of hexachlorophene in 15 kinds of fruits and vegetables (n = 6). (PDF)
