# Supplementary material for: Determination of hexachlorophene residue in fruits and vegetables by ultra-high performance liquid chromatography-tandem mass spectrometry
Source: PLoS One. 2024 Aug 14;19(8):e0307669. doi: 10.1371/journal.pone.0307669 (PMC11324096; doi:10.1371/journal.pone.0307669)
Supplement: S1 Fig — (A) acetonitrile-water, (B) methanol-water, (C) methanol-0.05% formic acid in water, (D) methanol-10 mmol/L ammonium acetate and 0.05% formic acid in water. (PDF) [file pone.0307669.s001.pdf]

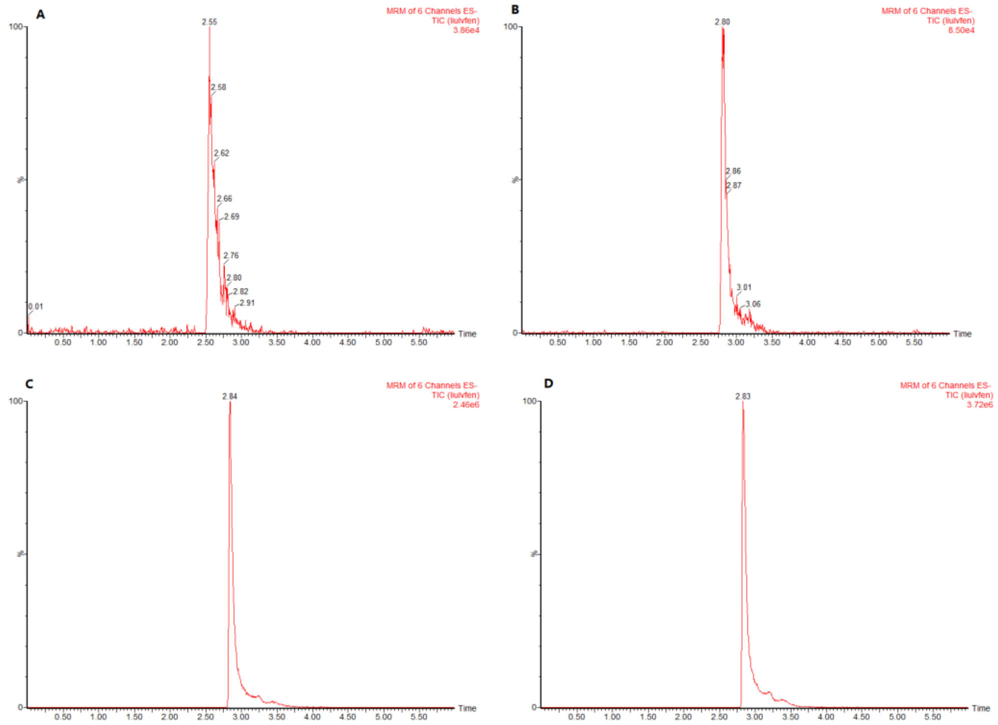

S1 Fig. TIC image of hexachlorophene standard solution using different mobile phases. (A) acetonitrile-water, (B) methanol-water, (C) methanol-0.05% formic acid in water, (D) methanol-10 mmol/L ammonium acetate and 0.05% formic acid in water.
