# Supplementary material for: Determination of hexachlorophene residue in fruits and vegetables by ultra-high performance liquid chromatography-tandem mass spectrometry
Source: PLoS One. 2024 Aug 14;19(8):e0307669. doi: 10.1371/journal.pone.0307669 (PMC11324096; doi:10.1371/journal.pone.0307669)
Supplement: S1 Table — (PDF) [file pone.0307669.s002.pdf]

S1 Table. Recoveries of standard solution after adsorption experiment with different adsorbent

No.	adsorbent	Recovery(%)			Average recovery (%)	standard deviation
		1	2	3		
1	Sillca	72.2	75.8	80.1	76.0	3.2
2	SAX	87.8	93.5	88.7	90.0	2.5
3	SCX	92.5	93.8	98.8	95.0	2.7
4	Al-N	94.7	102.9	93.3	97.0	4.2
5	Al-A	76.7	73.2	69.1	73.0	3.1
6	Al-B	91.4	87.3	85.2	88.0	2.6
7	NH2	31.1	26.5	38.2	31.9	4.8
8	GCB	27.1	31.6	37.1	31.9	4.1
9	PSA	26.8	36.1	23.9	28.9	5.2
10	C2	93	86.5	84.6	88.0	3.6
11	C8	87.3	81.7	77.3	82.1	4.1
12	C18	87.1	90.2	95.6	91.0	3.5
13	CN	77.8	81.9	89.2	83.0	4.7
14	PLS	14.3	19.4	29.3	21.0	6.2
15	PH	74.1	70.3	80.7	75.0	4.3
