# Supplementary material for: Determination of hexachlorophene residue in fruits and vegetables by ultra-high performance liquid chromatography-tandem mass spectrometry
Source: PLoS One. 2024 Aug 14;19(8):e0307669. doi: 10.1371/journal.pone.0307669 (PMC11324096; doi:10.1371/journal.pone.0307669)
Supplement: S2 Table — (PDF) [file pone.0307669.s003.pdf]

S2 Table. Recoveries of three matrix clean-up with different d-SPE adsorbents

Sample matrix.	adsorbent	Recovery(%)			Average recovery (%)	standard deviation
		1	2	3		
Potato	PSA	41.3	35.5	52.8	43.2	7.2
	GCB	62.8	69.2	72.9	68.3	4.2
	PSA+GCB	28.3	33.9	17.0	26.4	7.0
	Al-N	89.4	93.8	96.4	93.2	2.9
	SCX	95.2	90.3	100.6	95.4	4.2
	Al-N+ SCX	92.4	94.8	98.1	95.1	2.3
Tomato	PSA	78.4	84.3	72.0	78.2	5.0
	GCB	72.5	67.9	76.8	72.4	3.6
	PSA+GCB	41.7	47.1	53.0	47.3	4.6
	Al-N	92.2	87.5	94.2	91.3	2.8
	SCX	97.0	93.6	101.1	97.2	3.1
	Al-N+ SCX	93.1	99.9	96.0	96.3	2.8
Celery	PSA	91.5	83.4	92.7	89.2	4.1
	GCB	92.5	94.6	89.3	92.1	2.2
	PSA+GCB	72.5	65.7	78.8	72.3	5.3
	Al-N	89.0	94.5	85.3	89.6	3.8
	SCX	90.1	98.1	100.6	96.3	4.5
	Al-N+ SCX	90.6	88.7	82.0	87.1	3.7
