# Supplementary material for: Determination of hexachlorophene residue in fruits and vegetables by ultra-high performance liquid chromatography-tandem mass spectrometry
Source: PLoS One. 2024 Aug 14;19(8):e0307669. doi: 10.1371/journal.pone.0307669 (PMC11324096; doi:10.1371/journal.pone.0307669)
Supplement: S3 Table — (PDF) [file pone.0307669.s004.pdf]

S3 Table. The linear equations and correlation coefficient values of hexachlorophene

No.	Matrix	Regression equation	Correlation coefficient
1	acetonitrile	Y=14574.8 × X	0.999
2	Cabbage	Y=11719.2 × X	0.996
3	Celery	Y=13058.3 × X	0.996
4	Tomato	Y=12172.5× X	0.997
5	Eggplant	Y=14751.4 × X	0.997
6	Potato	Y=14748.5 × X	0.998
7	Radish	Y=14503.5 × X	0.996
8	Cowpea	Y=13076.7× X	0.997
9	Chives	Y=10815.3 × X	0.995
10	Apple	Y=13671.7× X	0.997
11	Peach	Y=13728.7 × X	0.998
12	Grape	Y=14386.2 × X	0.999
13	Citrus	Y=14983.5 × X	0.998
14	Bitter Melon	Y=13205.2 × X	0.996
15	Banana	Y=12943.6 × X	0.997
16	Hami Melon	Y=14896.2 × X	0.996
