# Supplementary material for: Determination of hexachlorophene residue in fruits and vegetables by ultra-high performance liquid chromatography-tandem mass spectrometry
Source: PLoS One. 2024 Aug 14;19(8):e0307669. doi: 10.1371/journal.pone.0307669 (PMC11324096; doi:10.1371/journal.pone.0307669)
Supplement: S4 Table — (PDF) [file pone.0307669.s005.pdf]

**S4 Table. The recoveries and relative standard deviations of hexachlorophene in 15 kinds of fruits and vegetables (n=6).**

| matrix. | Spiked level (μg/kg) | Recovery (%) | | | | | | Average recovery (%) | standard deviation |
|---|---|---|---|---|---|---|---|---|---|
| | | 1 | 2 | 3 | 4 | 5 | 6 | | |
| Cabbage | 2.0 | 99.1 | 84.0 | 93.4 | 79.0 | 96.4 | 79.2 | 88.5 | 9.2 |
| Cabbage | 4.0 | 80.3 | 91.1 | 92.6 | 77.5 | 85.3 | 87.5 | 85.7 | 6.3 |
| Cabbage | 20.0 | 86.1 | 94.4 | 90.7 | 83.0 | 91.3 | 90.9 | 89.4 | 4.2 |
| Celery | 2.0 | 77.4 | 76.3 | 84.1 | 82.9 | 92.6 | 99.1 | 85.4 | 9.5 |
| Celery | 4.0 | 89.4 | 94.7 | 74.3 | 79.9 | 76.1 | 93.8 | 84.7 | 9.8 |
| Celery | 20.0 | 78.7 | 82.5 | 87.4 | 86.7 | 80.2 | 85.5 | 83.5 | 3.9 |
| Tomato | 2.0 | 71.2 | 84.8 | 82.9 | 80.7 | 90.1 | 77.5 | 81.2 | 7.3 |
| Tomato | 4.0 | 78.5 | 73.0 | 80.3 | 79.9 | 87.9 | 81.5 | 80.2 | 5.5 |
| Tomato | 20.0 | 75.4 | 80.1 | 80.6 | 81.0 | 74.9 | 80.2 | 78.7 | 3.2 |
| Eggplant | 2.0 | 84.9 | 73.0 | 70.5 | 84.7 | 70.3 | 71.6 | 75.8 | 8.4 |
| Eggplant | 4.0 | 74.5 | 72.3 | 81.0 | 70.1 | 75.8 | 67.4 | 73.5 | 5.9 |
| Eggplant | 20.0 | 77.3 | 80.1 | 74.5 | 81.9 | 77.2 | 73.5 | 77.4 | 3.8 |
| Potato | 2.0 | 81.6 | 78.3 | 96.7 | 95.2 | 91.0 | 92.2 | 89.2 | 7.7 |
| Potato | 4.0 | 91.0 | 93.1 | 77.7 | 86.6 | 92.0 | 86.4 | 87.8 | 5.9 |
| Potato | 20.0 | 92.6 | 84.4 | 94.4 | 89.7 | 88.0 | 84.2 | 88.9 | 4.3 |
| Radish | 2.0 | 81.7 | 89.4 | 98.5 | 99.6 | 81.6 | 103.1 | 92.3 | 9.3 |
| Radish | 4.0 | 88.3 | 103.2 | 89.4 | 89.0 | 83.7 | 103.2 | 92.8 | 8.2 |
| Radish | 20.0 | 82.1 | 101.4 | 97.1 | 90.3 | 85.5 | 98.6 | 92.5 | 7.6 |
| Cowpea | 2.0 | 72.5 | 93.5 | 87.3 | 77.5 | 85.4 | 91.4 | 84.6 | 8.8 |
| Cowpea | 4.0 | 90.2 | 87.0 | 81.5 | 97.3 | 77.2 | 95.4 | 88.1 | 8.1 |
| Cowpea | 20.0 | 89.5 | 80.9 | 81.2 | 89.4 | 87.7 | 81.3 | 85.0 | 4.6 |
| Chives | 2.0 | 73.5 | 80.0 | 88.1 | 77.6 | 86.5 | 91.1 | 82.8 | 7.5 |
| Chives | 4.0 | 73.4 | 85.6 | 88.0 | 78.9 | 88.5 | 76.2 | 81.8 | 7.2 |
| Chives | 20.0 | 82.1 | 73.6 | 83.8 | 86.2 | 78.9 | 87.8 | 82.1 | 5.8 |
| Apple | 2.0 | 84.2 | 106.5 | 108.4 | 97.8 | 88.2 | 104.2 | 98.2 | 9.3 |
| Apple | 4.0 | 98.8 | 95.6 | 102.9 | 89.6 | 98.6 | 95.3 | 96.8 | 4.2 |
| Apple | 20.0 | 96.7 | 108.9 | 102.8 | 96.6 | 93.8 | 104.2 | 100.5 | 5.2 |
| Peach | 2.0 | 87.4 | 77.6 | 95.3 | 76.4 | 88.4 | 95.1 | 86.7 | 8.6 |
| Peach | 4.0 | 97.0 | 85.3 | 84.5 | 81.8 | 81.0 | 82.0 | 85.3 | 6.4 |
| Peach | 20.0 | 90.7 | 91.1 | 86.2 | 85.5 | 81.8 | 81.3 | 86.1 | 4.4 |
| Grape | 2.0 | 76.9 | 92.2 | 84.0 | 96.2 | 82.3 | 98.8 | 88.4 | 8.9 |
| Grape | 4.0 | 82.5 | 86.6 | 87.3 | 91.0 | 101.5 | 100.7 | 91.6 | 7.8 |
| Grape | 20.0 | 82.4 | 80.4 | 84.9 | 86.0 | 88.8 | 91.0 | 85.6 | 4.2 |
| Citrus | 2.0 | 68.3 | 71.9 | 66.5 | 76.5 | 70.1 | 87.7 | 73.5 | 9.6 |
| Citrus | 4.0 | 64.6 | 76.1 | 78.0 | 77.5 | 66.3 | 69.5 | 72.0 | 7.5 |
| Citrus | 20.0 | 81.2 | 77.2 | 68.1 | 80.8 | 85.8 | 71.3 | 77.4 | 7.8 |

| | | | | | | | | | |
|---|---|---|---|---|---|---|---|---|---|
| Bitter melon | 2.0 | 89.7 | 72.1 | 80.6 | 71.3 | 86.3 | 84.7 | 80.8 | 8.6 |
| Bitter melon | 4.0 | 89.6 | 71.8 | 85.3 | 75.8 | 82.5 | 83.3 | 81.4 | 7.3 |
| Bitter melon | 20.0 | 88.0 | 72.8 | 77.2 | 90.4 | 86.6 | 86.1 | 83.5 | 7.5 |
| Banana | 2.0 | 80.5 | 78.4 | 90.0 | 80.8 | 76.1 | 95.4 | 83.5 | 8.2 |
| Banana | 4.0 | 89.5 | 91.0 | 86.8 | 103.0 | 89.4 | 85.8 | 90.9 | 6.2 |
| Banana | 20.0 | 88.3 | 73.8 | 87.4 | 80.8 | 76.7 | 79.6 | 81.1 | 6.5 |
| Hami Melon | 2.0 | 78.7 | 79.1 | 69.2 | 87.8 | 69.5 | 87.4 | 78.6 | 9.5 |
| Hami Melon | 4.0 | 85.3 | 84.1 | 76.0 | 91.0 | 78.8 | 75.0 | 81.7 | 6.9 |
| Hami Melon | 20.0 | 81.0 | 82.6 | 85.0 | 85.7 | 76.7 | 73.2 | 80.7 | 5.5 |
